# A Rapid and Sensitive Detection Method for *Pseudomonas aeruginosa* Using Visualized Recombinase Polymerase Amplification and Lateral Flow Strip Technology

**DOI:** 10.3389/fcimb.2021.698929

**Published:** 2021-09-14

**Authors:** Haitao Yang, Yan Wang, Qiankun Yang, Hui Fan, Lei Wang, Tianmeng Zhang, Zhixing Li, Gang Liu, Panpan Zhao, Huahua Wu, Jingquan Dong, Wei Liang

**Affiliations:** ^1^Jiangsu Key Laboratory of Marine Biological Resources and Environment, Jiangsu Key Laboratory of Marine Pharmaceutical Compound Screening, Co-Innovation Center of Jiangsu Marine Bio-industry Technology, School of Pharmacy, Jiangsu Ocean University, Lianyungangg, China; ^2^Department of Laboratory Medicine, The Second People’s Hospital of Lianyungangg City, Lianyungangg, China; ^3^Key Laboratory of Zoonosis Research, Ministry of Education, College of Veterinary Medicine, Jilin University, Changchun, China; ^4^Laboratory Department of Ningbo First Hospital, Ningbo Hospital of Zhejiang University, Ningbo, China

**Keywords:** *Pseudomonas aeruginosa*, recombinase polymerase amplification, lateral flow strip, *elastase B*, cystic fibrosis

## Abstract

*Pseudomonas aeruginosa* is a common opportunistic pathogen that causes acute nosocomial necrotizing pneumonia and is the predominant source of chronic lung infections in patients with the genetic disorder cystic fibrosis. Early diagnosis in infected patients and monitoring *P. aeruginosa* contamination is therefore of great importance in controlling disease spread and development with timely drugs intervention treatment and cut off infection source. Traditional culture-biochemical methods are time consuming and highly dependent on technicians and expensive instruments. To address these challenges, the present study aimed to develop a rapid, sensitive, and specific, on-site detection method for *P. aeruginosa* based on recombinase polymerase amplification (RPA) combined with lateral flow strip (LFS) technology. The experimental process included screening and modification of primer and probe sets targeting the unique virulence gene *elastase B* (*lasB*); specificity detection in 29 strains of *P. aeruginosa* and 23 closely-related pathogenic bacteria; sensitivity measurements with gradient-diluted *P. aeruginosa* genomic DNA and probit regression analysis; and clinical application evaluation using 574 patients samples and calculating coincidence rate and kappa index value in comparison with the culture-biochemical method. The *P. aeruginosa* RPA-LFS assay could complete the amplification process at 37°C constant temperature within 30 min and results could be visualized by the naked eye within 10 min on LFS. The assay displayed high sensitivity with a limit of detection of 3.05 CFU/reaction. It also demonstrated high specificity by showing no cross reaction with other pathogenic bacteria, and rapidness by being completed in less than an hour. Furthermore, when used with clinical samples, the assay had a coincidence rate of 98.26% with the culture-biochemical method and a kappa index value of 0.9433. These data indicate that the RPA-LFS assay represents a major improvement for *P. aeruginosa* detection, especially in resource-limited areas.

## Introduction

*Pseudomonas aeruginosa* (*P. aeruginosa*) is a Gram-negative, aerobic, rod-shaped bacterium (Hilliam et al., 2020). It widely exists in air, soil, water (Crone et al., 2020), and food (Benie et al., 2017), and is a common opportunistic pathogen of immunocompromised individuals (Benie et al., 2017). Clinically, *P. aeruginosa* often causes skin and subcutaneous tissue infection, otitis media, meningitis, respiratory tract infection, urinary tract infection, sepsis, etc. Furthermore, *P. aeruginosa* is an important cause of acute nosocomial necrotizing pneumonia (Sadikot et al., 2005) and the predominant source of serious lung infections in patients with the genetic disorder cystic fibrosis (Winstanley et al., 2016). Antibiotic therapy were widely used in the clinical which as the primary treatment method, however, such infections in patients with cystic fibrosis are associated with high morbidity and mortality due to the significant antibiotic resistance of *P. aeruginosa* (Chatterjee et al., 2016; Pang et al., 2019). Thus, initial detection of *P. aeruginosa* in infected patients and monitoring *P. aeruginosa* contamination in drinking water and processed food are of great importance in controlling disease development with timely drug intervention and preventing disease occurrence by eliminating pathogen contamination risks.

The traditional culture-biochemical methods used in most hospitals are time-consuming, labor-intensive, and highly dependent on professional operation skills (Tramper-Stranders et al., 2005). Moreover, conventional culture diagnosis may fail to detect *P. aeruginosa* under a viable but non-culturable state in patients with cystic fibrosis (Mangiaterra et al., 2018). Serology-based immunological diagnosis methods, such as immunofluorescent assays and enzyme-linked immunosorbent assays, reduce the labor to some extent but are based on detection of specific antibodies, which are only generated a period of time after infection (Tramper-Stranders et al., 2005). Consequently, studies have focused on developing detection methods for *P. aeruginosa* based on molecular diagnostic techniques to shorten the detection period and simplify the operation process. However, most polymerase chain reaction (PCR)-based detection methods still rely on well-trained technicians and require precise machines, which restrict the application of such methods in remote areas and resource-limited laboratories (Granström et al., 1985; Tramper-Stranders et al., 2005; Shi et al., 2012; Mauch and Levy, 2014). Hence, there is an urgent need for a rapid, specific, sensitive, equipment-independent, on-site detection method for *P. aeruginosa*.

Development of the recombinase polymerase amplification (RPA) technique, which is independent of sophisticated laboratory instruments and specialist staff, has provided a molecular reference tool for detection of *P. aeruginosa* (James and Macdonald, 2015; Daher et al., 2016; Li et al., 2018). The RPA system solves the challenge of the traditional PCR amplification being dependent on a thermal cycler, and achieves amplification under a constant temperature between 37°C and 42°C (Jiang et al., 2020). First, a single-strand primer is combined by the recombinant enzyme UvsX to form stable nucleoprotein silk, which is employed to identify homologous sites paired to primer sequences in the template sequences and open the double-stranded DNA. Next, the single-strand binding protein gp32 binds to the single-strand DNA template and maintains double-complementary template sequences in a single-strand state. Meanwhile, UvsX recombinase is dissociated from the primer releasing the 3′-OH, and Bsu polymerase initiates amplification after recognition of the 3′-OH. With the aid of coenzyme UvsY, UvsX constantly binds to and separates from the primer making the amplification reaction continuous (Piepenburg et al., 2006; Lobato and O’Sullivan, 2018). Combining the gold nanoparticles (AuNPs)-coated lateral flow strip (LFS) with RPA to visually detect labeled amplification products further simplifies the detection process and realizes instrument-free, on-site detection (Dong et al., 2020).

The current study employed the promising RPA combined LFS technique to develop a rapid and sensitive on-site detection method for *P. aeruginosa*. Primer-probe sets for RPA assays were designed to target the highly specific virulence gene of *elastase B* (*lasB*) (Thibodeaux et al., 2007) in the strains of *P. aeruginosa* but not in other bacterial stains, and were screened in one standard strain of *P. aeruginosa* and 28 clinical isolates. Specificity of the assay was verified in nine respiratory bacterial pathogens and 14 food-borne or water-borne bacterial pathogens. Sensitivity of the RPA-LFS assay was measured in eight independent experiments and the limit of detection was 3.05 colony-forming units (CFU)/reaction. Finally, thee established RPA-LFS method for *P. aeruginosa* was evaluated in 574 clinical samples and the coincidence rate with the culture-biochemical method was 98.26% and the kappa index value was 0.9433. Overall, this study developed a rapid, sensitive, and specific RPA-LFS detection method for *P. aeruginosa* that has promising application in the initial point-of-care diagnosis in remote and resource-limited areas.

## Materials and Methods

### Ethics Statement

This study was approved by the Medical Ethics Committee of the Second People’s Hospital of Lianyungangg City (Permit Number: 2020005). For all collected clinical samples (from October 2020 to January 2021), such as sputum, vaginal discharge, wound discharge, etc., informed written consent, on an institutionally approved document, was provided by every patient prospectively following extensive discussions with each patient. All samples were de-identified, and their data presented in aggregate.

### Bacterial Strains and Clinical Samples

Standard strains of *P. aeruginosa, Aeromonas veronii, Aeromonas caviae, Aeromonas hydrophila, Aeromonas salmonicida, Salmonella typhimurium, Bacillus cereus, Yersinia enterocolitica, Staphylococcus aureus, Staphylococcus hominis, Staphylococcus epidermidis, Vibrio vulnificus, Vibrio alginolyticus, Vibrio cholerae and Vibrio parahaemolyticus* were preserved in our laboratory. A collection of 28 clinical isolates of P. aeruginosa and nine other common respiratory-isolated strains, including *Escherichia coli*, *Haemophilus influenzae, Acinetobacter baumannii*, *Klebsiella pneumoniae*, *Morganella fulton*, *Serratia marcescens*, *Burkholderia cepacia, Bacillus mirabilis*, and *Streptococcus pneumoniae* were kindly provided by the Second People’s Hospital of Lianyungangg City (Jiangsu, China). All strains were identified by reference culture-biochemical methods in the Department of Laboratory Medicine, the Second People’s Hospital of Lianyungangg. Bacterial strains used in the study are listed in **Table 1**.

**Table 1:** Bacterial strains used in the study.

Species	Source	Strain Amount	Origin/Designation	Culture-biochemical Methods	RPA-LFS
*Pseudomonas aeruginosa*	Reference strain	1	ATCC 27853	Positive	Positive
*P. aeruginosa*	Sputum isolated strain	28	Lianyungang, China	Positive	Positive
*Escherichia coli*	Sputum isolated strain	1	Lianyungang, China	Negative	Negative
*Haemophilus influenzae*	Sputum isolated strain	1	Lianyungang, China	Negative	Negative
*Acinetobacter baumannii*	Sputum isolated strain	1	Lianyungang, China	Negative	Negative
*Klebsiella pneumoniae*	Sputum isolated strain	1	Lianyungang, China	Negative	Negative
*Morganella Fulton*	Sputum isolated strain	1	Lianyungang, China	Negative	Negative
*Serratia marcescens*	Sputum isolated strain	1	Lianyungang, China	Negative	Negative
*Burkholderia cepacian*	Sputum isolated strain	1	Lianyungang, China	Negative	Negative
*Bacillus mirabilis*	Sputum isolated strain	1	Lianyungang, China	Negative	Negative
*Streptococcus pneumoniae*	Sputum isolated strain	1	Lianyungang, China	Negative	Negative
*Aeromonas veronii*	Reference strain	1	BNCC138468	Negative	Negative
*Aeromonas caviae*	Reference strain	1	ATCC15468	Negative	Negative
*Aeromonas hydrophila*	Reference strain	1	ATCC 35654	Negative	Negative
*Aeromonas salmonicida*	Reference strain	1	ATCC 33658	Negative	Negative
*Salmonella typhimurium*	Reference strain	1	ATCC 14028	Negative	Negative
*Bacillus cereus*	Reference strain	1	ATCC 14579	Negative	Negative
*Yersinia enterocolitica*	Reference strain	1	ATCC 23715	Negative	Negative
*Staphylococcus aureus*	Reference strain	1	ATCC 6538	Negative	Negative
*Staphylococcus hominis*	Reference strain	1	ATCC 27844	Negative	Negative
*Staphylococcus epidermidis*	Reference strain	1	ATCC 35984	Negative	Negative
*Vibrio vulnificus*	Reference strain	1	ATCC 29307	Negative	Negative
*Vibrio alginolyticus*	Reference strain	1	ATCC 17749	Negative	Negative
*Vibrio cholerae*	Reference strain	1	ATCC 14100	Negative	Negative
*Vibrio parahemolyticus*	Reference strain	1	ATCC 17802	Negative	Negative

ATCC, American Type Culture Collection, Rockville, Maryland, USA; BNCC, BeNa Culture Collection, Beijing, China.

A collection of 574 clinical samples, including 491 sputum samples, 27 vaginal discharge samples, 15 wound discharge samples, 28 oral secretion samples, 7 urine samples, 4 blood samples, and 2 alveolar lavage fluid samples collected from patients were kindly provided by the Second People’s Hospital of Lianyungangg.

### DNA Preparation

RPA could tolerate crude DNA samples during amplification (Silva et al., 2018). For DNA extraction from standard strains and isolated strains, 1 mL bacterial cultures were centrifuged at 12,000 ×*g* for 1 min and the resulting pellets were resuspended in 50 μL TE buffer. The enriched bacterial suspension was inactivated at 100°C for 10 min to release genomic DNA. DNA extraction from clinical samples was achieved using the QIAamp DNA Microbiome Kit (QIAGEN, Germany) according to the manufacturer’s instructions.

### Design of RPA Primer and Probe

Specific RPA primer sets based on *lasB* gene sequences (GenBank no. CP011857.1) were designed using the National Center for Biotechnology Information (NCBI) Primer-BLAST online designing software. A specific RPA probe was designed using Primer Premier 3 software based on sequences where screened RPA primers performed well. The parameters of primer and probe design were set referring to previously described parameters (Wu et al., 2020). The reverse primer for RPA-LFS was labeled with biotin at the 5′ end. The probe for RPA-LFS was labeled with fluorescein isothiocyanate (FITC) at the 5′ end, blocked with a C3 spacer at the 3′ end, and a “C” base was replaced with a tetrahydrofuran (THF) group in the middle of the sequence. All the primers and probes were synthesized by General Biol (Anhui, China) and listed in **Tables 2**–**4**.

**Table 2:** Design of specific RPA primer sets.

Primer sets	Description	Sequence (5’-3’)	Amplicon size (bp)
#1	F1	ACTGAACTAGATGAAGAAGGTTTCTA	456
	R1	CAGTTCCACTTTGTCATTCTCGGTCTTG	
#2	F1	ACTGAACTAGATGAAGAAGGTTTCTA	
	R2	AGTTCCACTTTGTCATTCTCGGTCTTG	
#3	F1	ACTGAACTAGATGAAGAAGGTTTCTA	
	R3	GTTCCACTTTGTCATTCTCGGTCTTG	
#4	F4	GTTTCTACGCTTGACCTGTTGTTCGTTG	275
	R4	GGACCCTTGACTTCGGTGATGGCTT	
#5	F5	ATGTTCTATCCGCTGGTGTCGCTGGA	204
	R5	ACCGCTGCCCTTCTTGATGTCGTAG	

F and R represent forward and reverse primers, respectively.

**Table 3:** Design and modification of RPA-LFS probe and reverse primer sets..

Description	Sequence (5’-3’)
**Probe**	FITC-GATGAAGAAGGTTTCTACGCTTGTCCTGTT[THF]TTCGTTGCTATTAT-C3 Spacer
**R1-m**	Biotin-CAGTTCCACTTTGTCATTCTCGGTC
R2-m	Biotin-AGTTCCACTTTGTCATTCTCGGTTTTG
R3-m	Biotin-GTTCCACTTTGTCATTCTCGGTTTTG

Mismatched bases are highlighted in red. F and R represent forward and reverse primers, respectively, and m indicates modification.

**Table 4:** Modification of RPA-LFS forward primers.

Description	Sequence (5’-3’)
F1-m1	ACTGAACTAGATGAAGCAGGTTTTTA
F1-m2	ACTGAAGTAGATGAAGATGGTTTATA
**F1-m3**	ACTGATCTAGATGAAGATGGTTTAT

Mismatched bases are highlighted in red. F represents forward primer and m indicates modification.

### RPA Procedure

For initial screening of forward and reverse primer sets, RPA amplification was conducted using a TwistAmp Liquid DNA Amplification Kit (TwistDx, UK) according to the manufacturer’s instructions. Each 50 μL mixture contained 25 μL reaction buffer, 11.9 μL double-distilled H_2_O (ddH_2_O), 5 μL Basic e-mix, 2.5 μL core mix, 2.1 μL RPA forward and reverse primers (10 μM), and 1 μL genomic DNA. The reaction was initiated by adding 2.5 μL of 280 mM Mg(Ac)_2_ to the mixture and immediately incubating at 37°C for 30 min. Amplification products were purified with Universal DNA Purification Kit (TIANGEN, Beijing) and were detected *via* 1.5% agarose gel electrophoresis.

### RPA-LFS Procedure

To screen the combination of probe and primer sets, RPA-LFS assays were conducted with a TwistAmp DNA Amplification nfo Kit (TwistDx, UK) according to the manufacturer’s instructions. Each 50 μL reaction system was composed of 29.5 μL rehydration buffer, 14.3 μL ddH_2_O, 2.1 μL RPA forward and reverse primers (10 μM), 1 μL genomic DNA, 0.6 μL RPA probe (10 μM), and a dried enzyme pellet. The enzyme complex was activated by adding 2.5 μL of 280 mM Mg(Ac)_2_ to the mixture. After centrifugation at 2,000 ×*g* for 1 min, the reaction was immediately incubated at 37°C for 30 min. Resulting amplification products were detected by LFS (Ustar Biotechnologies, Hangzhou, China) by loading 5 μL reaction mixture into the sample pad of the LFS and inserting into 100 μL sample buffer for 5 min. Results were readable by the naked eye.

### Specificity Assay

The specificity of the RPA-LFS for *P. aeruginosa* was evaluated using genomic DNA from nine respiratory bacterial pathogens, 14 food-borne or water-borne bacterial pathogens, and 28 clinical isolates of *P. aeruginosa* mentioned in the Bacteria Strains section.

### Limit of Detection (LOD) Assay

A 10-fold series dilution of inactivated cultures of *P. aeruginosa* were prepared with final concentrations ranging from 5 × 10^4^ CFU/mL to 5 × 10^0^ CFU/mL and were dissolved in 50 μL TE buffer after centrifugation at 2,000 ×*g* for 1 min. The LOD was determined by probit regression analysis from eight independent experiments.

### Evaluation of the Coincidence Rate of RPA-LFS With the Culture-Biochemical Method Using Clinical Samples

The detection coincidence rate of the RPA-LFS assay in clinical samples was evaluated by comparison with the traditional culture-biochemical method. Clinical samples were cultured on selective media, including blood agar, chocolate agar, and MacConkey agar at 37°C for 18-48 h. Bacterial identification was performed with a VITEK 2 machine (bioMérieux, France) and additional biochemical tests if necessary. The coincidence rate between two methods was calculated by the following formulas: {(number of positive samples in both of the two assays + number of negative samples in both of the two assays)/total number of samples} × 100%. The kappa index value was determined to evaluate this assay.

### Statistical Analysis

Gray values were generated using ImageJ software and differences among multiple groups were analyzed by one-way analysis of variance (ANOVA) using SPSS 22.0 (Chicago, IL, USA) with results expressed as the mean ± SEM. The homogeneity of variance of the data was analyzed by Levene’s test. If homogeneity of variance was identified, the data were analyzed using the post-hoc Bonferroni test (B), while if heterogeneity of variance was identified, the data were analyzed using Tamhane’s T2 test (M). Graphs were generated in GraphPad Prism 7.00 (GraphPad Software Inc., La Jolla, CA, USA). Significance is indicated by different lowercase letters (p<0.05). Probit regression analysis from eight independent experiments was performed using SPSS software to determine the LOD. Kappa index values, determined using SPSS software, were used to estimate the coincidence of results between the RPA-LFS and culture-biochemical method.

## Results

### Design and Initial Screening of RPA Primers for *P. aeruginosa*

**Figure 1:**
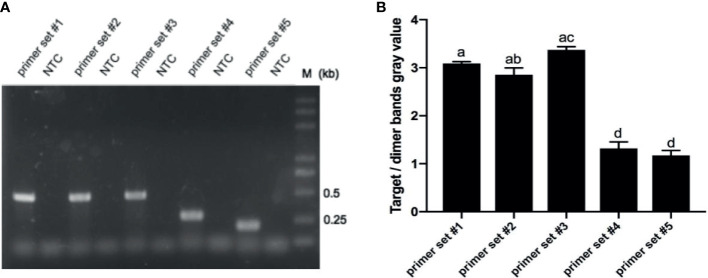
RPA primer sets screen. **(A)** Primer sets of #1-5 were screened using RPA assays with *P. aeruginosa* standard strain genomic DNA as templates. A no template control (NTC) for each primer set was used as a negative control. Equal volumes of amplification products (5 μL) were detected on 1.5% agarose gel through electrophoresis. **(B)** Gray values of target/dimer bands were measured using ImageJ software by measuring the parameters of gray value and area, and calculated using Excel software. Results are mean ± SEM of triplicate experiments. Bars with different lowercase letters are significantly different (*p*<0.05) while bars with identical lowercase letters are not significantly different (*p*>0.05). Samples are indicated on the top of the images.

Rational design of primers for the detection of *P. aeruginosa* commenced with a Primer BLAST search on the highly conservative virulence gene sequences of *lasB*. The primer sets only paired with *P. aeruginosa* and not any other bacterial stains. As shown in **Table 1**, five pairs of primer sets targeting to three areas of the *lasB* gene were obtained. Screening of the initial primer sets was performed using genomic DNA of standard strains of *P. aeruginosa* as templates in the RPA assay and the resulting products were detected by agarose gel electrophoresis. All five primer sets from #1 to #5 generated clear targeted bands with sizes of 456, 456, 456, 275, and 204 bp, respectively. Though no unspecific amplification bands existed in the no template control (NTC), primer-dimers smaller than 100 bp occurred unavoidably in the experimental groups (**Figure 1A**). Thus, gray values of the target/dimer bands were further analyzed. RPA amplification with primer sets #1, #2, and #3 displayed high gray values of target/dimer bands (**Figure 1B**), while gray values in primer sets #4 and #5 were both significantly lower than those of primer sets #1, #2, and #3 (p<0.05). Thus, primer sets #1, #2, and #3 were selected for further screening of RPA-LFS primers and probes.

### Modification and Determination of the Optimal Combination of Primers and Probe for RPA-LFS

**Figure 2:**
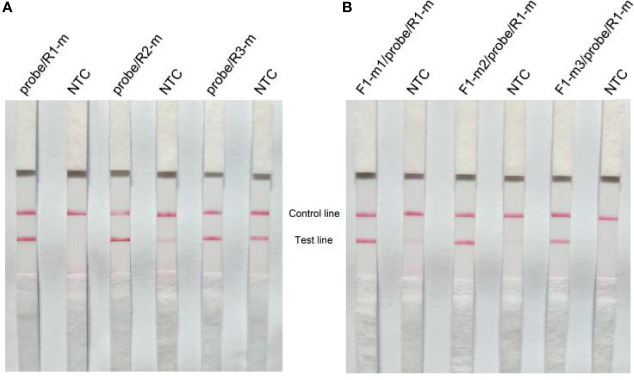
RPA-LFS primer and probe sets screen. **(A)** Probe-reverse primer sets of probe/R1-m, probe/R2-m, and probe/R3-m were screened using RPA assays with genomic DNA from standard strains of *P. aeruginosa* as the templates. A no template control (NTC) for each primer set was used as the negative control. Equal volumes of amplification products (5 μL) were detected using LFS. **(B)** Forward primer-probe/R1-m sets of F1-m1/probe/R1-m, F1-m2/probe/R1-m, F1-m3/probe/R1-m were screened using RPA assays with genomic DNA of standard strains of *P. aeruginosa* as the templates. A NTC for each primer set was used as the negative control. Equal volumes of amplification products (5 μL) were detected using LFS. m labels in the primers represents modification; F and R indicates forward and reverse primers, respectively. Samples are indicated on the top of the strips.

LFS can specially recognize FITC- and biotin-labeled RPA products generated by probe and reverse primer. Thus, the NTC signal should be totally inhibited when designing a specific probe for RPA-LFS. Previous studies demonstrated that the RPA reaction could tolerate a few mismatches between the primer/probe and the template (Daher et al., 2015; Wu et al., 2020). As shown in **Table 3**, probe-reverse primer dimers were analyzed using Primer Premier 3 software and mismatches were added (indicated by red text in **Table 3**) to break sites that had more than three successive bases or more than one base at the 3′ end. The revised reverse primers were named R1-m, R2-m, and R3-m, respectively. RPA-LFS assays were then conducted using the probe and modified reverse primers. Positive signals at the test line occurred in all probe/modified reverse primer groups when amplifying the *lasB* gene using genomic DNA of *P. aeruginosa* (**Figure 2A**). However, false-positive signals occurred in the NTC group reactions except for those using the probe and R1-m reverse primer combination. Next, the forward primer F1 was modified according to the principles described above and resulting sequences are listed in **Table 4**. The combination of F1-m3/Probe/R1-m used in the RPA-LFS assays for detection of *P. aeruginosa* displayed no signals at the test line in the NTC group and distinct signals at the test line in the group containing *P. aeruginosa* genomic DNA (**Figure 2B**). Overall, the optimal primer-probe set of F1-m3/Probe/R1-m was obtained for RPA-LFS detection of *P. aeruginosa*.

### Specificity Analysis of the RPA-LFS Assay

**Figure 3:**
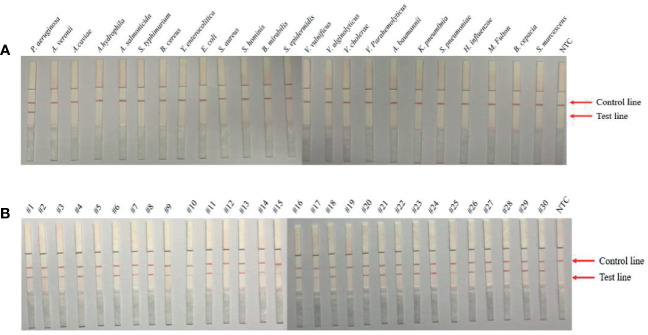
Specificity analysis of *P. aeruginosa* RPA-LFS assay. The specificity of the established RPA-LFS detection system for *P. aeruginosa* was tested using genomic DNA extracted from 9 common respiratory bacterial pathogens or 14 food-borne or water-borne bacterial pathogens **(A)** and 28 clinical isolates of *P. aeruginosa* **(B)**. A no template control (NTC) was used as the negative control, and *P. aeruginosa* was used as the positive control. RPA amplification results were detected using LFS and samples are indicated on the top of the strips.

During the design process of the primer sets, parameters were restricted to *P. aeruginosa*. However, to evaluate the specificity of the optimal F1-m3/Probe/R1-m set for RPA-LFS detection of *P. aeruginosa*, a total of 23 common respiratory bacterial pathogens or food-borne or water-borne bacterial pathogens and 28 clinical isolates of *P. aeruginosa* were used. As shown in **Figure 3A**, a marked positive signal occurred at the test line when using *P. aeruginosa* genomic DNA as the template, but in contrast, no bands were present at the test lines when using genomic DNA from other respiratory bacterial pathogens and food-borne or water-borne bacterial pathogens as the template. Furthermore, all 28 isolated strains of *P. aeruginosa* showed positive signals in the RPA-LFS assay (**Figure 3B**). Overall, these results illustrated that the established RPA-LFS detection system had good specificity towards *P. aeruginosa* and no cross reaction with other pathogenic bacteria.

### LOD Measurement of the RPA-LFS Assay

**Figure 4:**
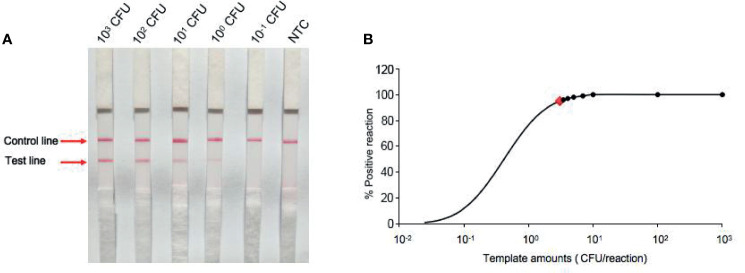
Determination of limit of detection (LOD) of *P. aeruginosa* RPA-LFS assay. **(A)** The LOD of the established RPA-LFS detection system for *P. aeruginosa* was determined from eight independent assays using genomic DNA from *P. aeruginosa* in a serial dilution ranging from 10^-1^ to 10^3^ CFU per reaction. Images were the RPA-LFS results and template amounts are indicated on the top of the strips. **(B)** Probit regression analysis using SPSS software was conducted on data collected from eight repeats.

The detection limit of the RPA-LFS assay was evaluated with templates of a 10-fold series dilution of inactivated *P. aeruginosa* culture from 10^-1^ to 10^3^ CFU (in 1 μL for a 50-μL RPA reaction volume). Strong signals occurred at the test lines in the 10^3^ CFU group and the signals became weaker with the decrease in template concentrations and had disappeared in the 10^-1^ CFU group (**Figure 4A**). Moreover, not all assays produced positive results when using templates of 10^0^ (6 positive results out of 8 samples, 6/8) and 10^-1^ (1/8) CFU amounts. To further determine the accurate LOD of the RPA-LFS assay, a probit regression analysis was conducted using statistical analysis *via* SPSS software on data from eight independent assays. The LOD was 3.05 CFU per reaction at 95% probability (**Figure 4B**).

### Application of *P. aeruginosa* RPA-LFS in Clinical Samples Detection

To evaluate the clinical application of the established RPA-LFS system for detecting *P. aeruginosa*, a total of 574 clinical samples from patients were collected by the clinical laboratory of the Second People’s Hospital of Lianyungangg City and were tested in the RPA-LFS assay and by culture-biochemical methods. As shown in **Table 5**, 112 of the 574 samples were positive for *P. aeruginosa* using the RPA-LFS assay, whereas 103 of the 574 samples were positive by the culture-biochemical method. Among the 574 samples, 103 were positive and 461 were negative in both detection methods. The coincidence rate of the established RPA-LFS detection assay with the traditional culture-biochemical method was 98.26%. The calculated kappa index value was 0.9433, which illustrated there was no statistical significance between these two methods (p>0.05). These results indicated the feasibility and reliability of the highly specific and sensitive *P. aeruginosa* RPA-LFS when applied to clinical samples from patients.

**Table 5:** Determination of coincidence rate between RPA-LFS assay and culture-biochemical methods in clinical samples.

		RPA-LFS assay		Total
		Positive	Negative	
**Culture-biochemical method**	**Positive**	103	0	103
**Negative**	9	461	470
**Total**		112	461	574

## Discussion

*P. aeruginosa*, an important opportunistic pathogen, can increase the risk of morbidity and mortality in patients with cystic fibrosis (Lund-Palau et al., 2016). Early detection and appropriate treatment would prevent or reduce chronic colonization with *P. aeruginosa* (Li et al., 2005; Kidd et al., 2015). Molecular diagnosis techniques for *P. aeruginosa* that display higher sensitivity than traditional time-coursing culture-based detection methods have been developed. However, there are difficulties with these molecular diagnosis methods in that they require sophisticated instruments and well-trained technicians, which restrict their application in resource-limited areas. In the current study, a fast and specific, on-site detection method for *P. aeruginosa* was established based on an RPA combined LFS technique; these assay characteristics are essential for the effective control of *P. aeruginosa* outbreaks. Considering the antibiotic resistance in the CF patients (Ang et al., 2016), this method was the first step that provided rapid results in the clinical diagnosis, and the traditional culture-biochemical method was still need to screen which antibiotics were sensitive to, and determine the drugs of treatment in the follow therapy.

An extensive array of secreted proteins constitute the major components of *P. aeruginosa* virulence, and several virulence factors that contribute to the damage induced by this opportunistic pathogen in humans have been identified, including surface structures (Miao et al., 2007), extracellular products (Traidej et al., 2003; Lau et al., 2004; Spencer et al., 2010; Park et al., 2014), and a type III secretion system (Sato et al., 2003; Barbieri and Sun, 2004; Galle et al., 2012). *LasB*, an important extracellular virulence factor that belongs to the protease category of enzymes, plays a crucial role in the pathogenicity of *P. aeruginosa* during infection (Thibodeaux et al., 2007). Moreover, *lasB* was the most frequent virulence gene identified in screens for virulence factors in patients of Northwestern Morocco (98.7%), children with cystic fibrosis in Iran (95.4%), patients with community- and hospital-acquired diarrhea (98%), and mineral water and spring water in China (100%) (Faraji et al., 2016; Elmouaden et al., 2019; Fakhkhari et al., 2020; Wei et al., 2020). Considering the wide distribution of *lasB*, this virulence gene was selected as the target for the assay developed in the current study.

Screening of primer and probe sets is critical in establishing a successful RPA-LFS detection method for *P. aeruginosa*. Two principles for identifying suitable primer and probe sets were followed in the current study. The first was that positive signals should only occur when using *P. aeruginosa* as a template and not the NTC, while the second principle was that the detection method should only recognize *P. aeruginosa*, not other common, closely related bacterial pathogens. Based on the recommended advice for design of RPA primers, five primer sets targeting three conservative areas of the *lasB* gene were selected and the products resulting from RPA amplification were detected by agarose gel electrophoresis. Gray values were used to select primer sets with higher amounts of target products and less primer dimers. To enable visual detection of products on LFS, labeled probes were introduced into the RPA reaction and a mismatch was engineered in the primers to guarantee the NTC was controlled after probe/reverse primer sets and forward/probe-reverse screening.

Specificity evaluation was made based on 28 isolated strains of *P. aeruginosa*, nine respiratory bacterial pathogens (including *E. coli*, *H. influenzae*, *A. baumannii*, *K. pneumoniae*, *M. fulton*, *S. marcescens*, *B. cepacia*, *B. mirabilis*, and *S. pneumoniae*), and 14 food-borne or water-borne bacterial pathogens (including *A. veronii*, *A. caviae*, *A. hydrophila*, *A. salmonicida*, *S. typhimurium*, *B. cereus*, *Y. enterocolitica*, *S. aureus*, *S. hominis*, *S. epidermidis*, *V. vulnificus*, *V. alginolyticus*, *V. cholerae*, and *V. parahaemolyticus*). The isolates of *P. aeruginosa* were collected from the most frequent sputum samples of patients infected with *P. aeruginosa*; the patients were different ages and genders. The established RPA-LFS assay was compatible with detection of these isolated strains of *P. aeruginosa*. Furthermore, there was no cross-reaction with respiratory pathogens often found in co-infections with *P. aeruginosa* or with other commonly detected clinical pathogens when using the optimal RPA-LFS primer-probe set. This illustrated that the established *P. aeruginosa* RPA-LFS had good specificity and could be used to determine the presence of *P. aeruginosa* during diagnosis.

It is recognized that enhanced sensitivity is another key factor in the development of diagnosis methods. To determine the accurate LOD of *P. aeruginosa* RPA-LFS, different template amounts of *P. aeruginosa* genomic DNA ranging from 10^3^ CFU to 10^-1^ CFU per reaction were tested and results were evaluated from eight independent assays. With probit regression analysis, the LOD of the *P. aeruginosa* RPA-LFS was 3.05 CFU per reaction at 95% probability and this was comparable with the LOD of other highly sensitive molecular detection methods, including multiplex PCR (10^3^ CFU or 5 pg), single PCR with purified DNA (0.2 pg) or crude bacterial culture (3 CFU), and loop-mediated isothermal amplification (3.25 CFU) (Song et al., 2000; da Silva Filho et al., 2004; Goto et al., 2010; Gan et al., 2020). In the clinical application, such sensitive RPA-LFS assay for *P. aeruginosa* faces obstacles of false positive issues, which was attributed to the aerosol pollution in the environment. In order to avoid such cases, strategies of nucleic acid clearance was necessary to be used to avoid aerosol contamination in the reaction and detection area. Evaluation of the RPA-LFS assay for on 574 clinical samples of sputum, vaginal discharge, wound discharge, oral secretion, urine, blood, and alveolar lavage fluid revealed a coincidence rate of 98.26% with the traditional culture-biochemical method, and a kappa index value of 0.9433. The highly consistent results between these two methods demonstrated that the *P. aeruginosa* RPA-LFS assay showed promising clinical application, especially in instrument-limited areas.

In conclusion, a rapid, specific, and sensitive, on-site detection assay for *P. aeruginosa* was established based on visualized RPA combined LFS technologies targeting to the *lasB* virulence gene. Readable results could be obtained within one hour *via* an easy operation process and equipment-free conditions. The assay has a promising prospect for onsite detection in resource-limited areas.

## Data Availability Statement

The original contributions presented in the study are included in the article/supplementary material. Further inquiries can be directed to the corresponding authors.

## Ethics Statement

The studies involving human participants were reviewed and approved by the Medical Ethics Committee of the Second People’s Hospital of Lianyungangg City. The patients/participants provided their written informed consent to participate in the study.

## Author Contributions

JD, WL and HW designed the research. HY, YW, QY and HW conducted the research. HY, YW, QY, HF, LW, TZ, ZL, GL and PZ analyzed the data. QY and HY wrote the manuscript. JD and WL directed the project. All authors contributed to the article and approved the submitted version.

## Funding

The authors thank the Natural Science Foundation of Jiangsu Province (BK20191210), the Open Foundation of Key Jiangsu Institute of Marine Resources Development (JSIMR202016), the fifth phase of “333 Project” scientific research project in Jiangsu Province (BRA2019248), the Subject of Lianyungangg Science and Technology Bureau (SF2015), the Jiangsu Distinguished Professor program (KK19515), and the Priority Academic Program Development of Jiangsu Higher Education Institutions of China for financial support. The funders had no role in study design, data collection and analysis, decision to publish, or preparation of the manuscript.

## Conflict of Interest

The authors declare that the research was conducted in the absence of any commercial or financial relationships that could be construed as a potential conflict of interest.

## Publisher’s Note

All claims expressed in this article are solely those of the authors and do not necessarily represent those of their affiliated organizations, or those of the publisher, the editors and the reviewers. Any product that may be evaluated in this article, or claim that may be made by its manufacturer, is not guaranteed or endorsed by the publisher.
